# The sonication-assisted whisker method enables CRISPR-Cas9 ribonucleoprotein delivery to induce genome editing in rice

**DOI:** 10.1038/s41598-023-40433-w

**Published:** 2023-09-07

**Authors:** Akiyoshi Nakamura, Tsubasa Yano, Nobutaka Mitsuda, Maiko Furubayashi, Seiichiro Ito, Shigeo S. Sugano, Teruhiko Terakawa

**Affiliations:** 1 Bioproduction Research Institute, National Institute of Advanced Industrial Science and Technology (AIST), 1-1-1, Higashi, Tsukuba, Japan; 2 Inplanta Innovations Inc., 4-5-11, Namamugi, Tsurumi-ku, Yokohama, Japan; 3 Bioproduction Research Institute, National Institute of Advanced Industrial Science and Technology (AIST), Sapporo, Japan; 4 TOPPAN INC, Tokyo, Japan

**Keywords:** Plant biotechnology, Plant breeding

## Abstract

CRISPR/Cas9-based genome editing represents an unprecedented potential for plant breeding. Unlike animal cells, plant cells contain a rigid cell wall, genome editing tool delivery into plant cells is thus challenging. In particular, the delivery of the Cas9-gRNA ribonucleoprotein (RNP) into plant cells is desired since the transgene insertion into the genome should be avoided for industrial applications in plants. In this study, we present a novel RNP delivery approach in rice. We applied the sonication-assisted whisker method, conventionally developed for DNA delivery in plants, for RNP delivery in rice. Combined with marker gene delivery, we successfully isolated *OsLCYβ* genome-edited lines generated by RNPs. The calli and regenerated shoot of the ﻿*OsLCYβ* mutant showed abnormal carotenoid accumulation. In addition, we also detected, although at a low frequency, genome editing events in rice calli cells by RNP delivery using the sonication-assisted whisker method without any additional. Therefore, the sonication-assisted whisker method could be an attractive way to create RNP-based genome-edited lines in plants.

## Introduction

Plant breeding has entered a new era with the invention of the clustered regularly interspaced short palindromic repeats (CRISPR)-associated protein 9 (Cas9)-based genome editing system and its subsequent improvements^1^. Ribonucleoproteins (RNPs) of the *Streptococcus pyogenes* Cas9 (SpCas9) protein with a single guide RNA (gRNA) specifically bind to the DNA harboring gRNA target sequence followed by the protospacer adjacent motif (PAM) 5′-NGG-3′ sequence. RNPs induce double-strand breaks (DSB) at the DNA target site. The programmable feature of the CRISPR technology displays relevant potential in genome breeding. The DNA fragment that encodes the CRISPR-Cas9 components, Cas9 and gRNA, is usually large, over 7 kbp long including promoter or terminators. To lower the chances of this large-size DNA randomly integrating into the genome, it is important to directly deliver the RNP. Unlike DNA delivery by *Agrobacterium*, RNP cannot easily be delivered to the plant cells across the cell wall. Accordingly, RNP delivery technology development gathers attention recent years^2^.

The delivery method for RNA encoding CRISPR-Cas9 or RNP in plants have been developed intensively^3–6^. Currently, the PEG^7, 8^ and the particle bombardment^9^ methods are the two major ways of introducing RNPs into plant cells. However, the former requires the isolation of protoplasts, from which regenerating individuals is generally difficult^6^. The particle bombardment method allows for a relatively easier regeneration of plant individuals than the PEG method since it usually targets intact totipotent cells (e.g., those in the callus or shoot apical meristem). However, the RNP introduction efficiency is generally low^9, 10^, partially due to the small number of RNP molecules that could be introduced into the cells. Moreover, during microinjectile preparation, the RNPs in the buffer should be dried, potentially leading to the inactivation of the Cas9 endonuclease activity. Therefore, the development of new methods for large-scale RNP introduction into plant cells retains relevant research interest.

The whisker method is a gene delivery approach in plants using high aspect-ratio silicone-carbide or aluminum borate whiskers, which could penetrate the cell wall and membrane^11, 12^. Whiskers premixed with plasmids directly pierce the plant cell in solution without any dry-up process. This simple introduction using whiskers allows for less species-dependent gene delivery into plant cells. Several studies reported transgenic plant generation using the whisker method in rice, maize, cotton, and others^13–16^. We previously published a significantly improved version of the whisker method, the sonication-assisted whisker transformation, coined the whisker-supersonic method (WSS) in rice^17, 18^. The WSS approach uses sonication to introduce the whiskers into the plant cells. Using this method, transgenic soybeans have been efficiently developed^18, 19^. This method was also applicable to the tree species *Cryptomeria japonica*^20^. However, no report has demonstrated its applicability to deliver proteins into plant cells.

In this study, we demonstrate the development of the sonication-assisted whisker-based RNP delivery and genome editing in rice. We successfully identified the RNP-delivered calli with mutations at the target genes with the co-transfection of a selection marker-harboring plasmid. We disrupted the *OsLCYβ* gene in rice using this method and demonstrated that it led to abnormal carotenoid accumulations both in the genome-edited calli and regenerated individuals with practical efficiency. Taken together, the sonication-assisted whisker method could be a feasible and attractive tool for RNP-based genome editing in plants.

## Results

### RNP delivery using the whisker method and genome editing event identification

**Figure 1:**
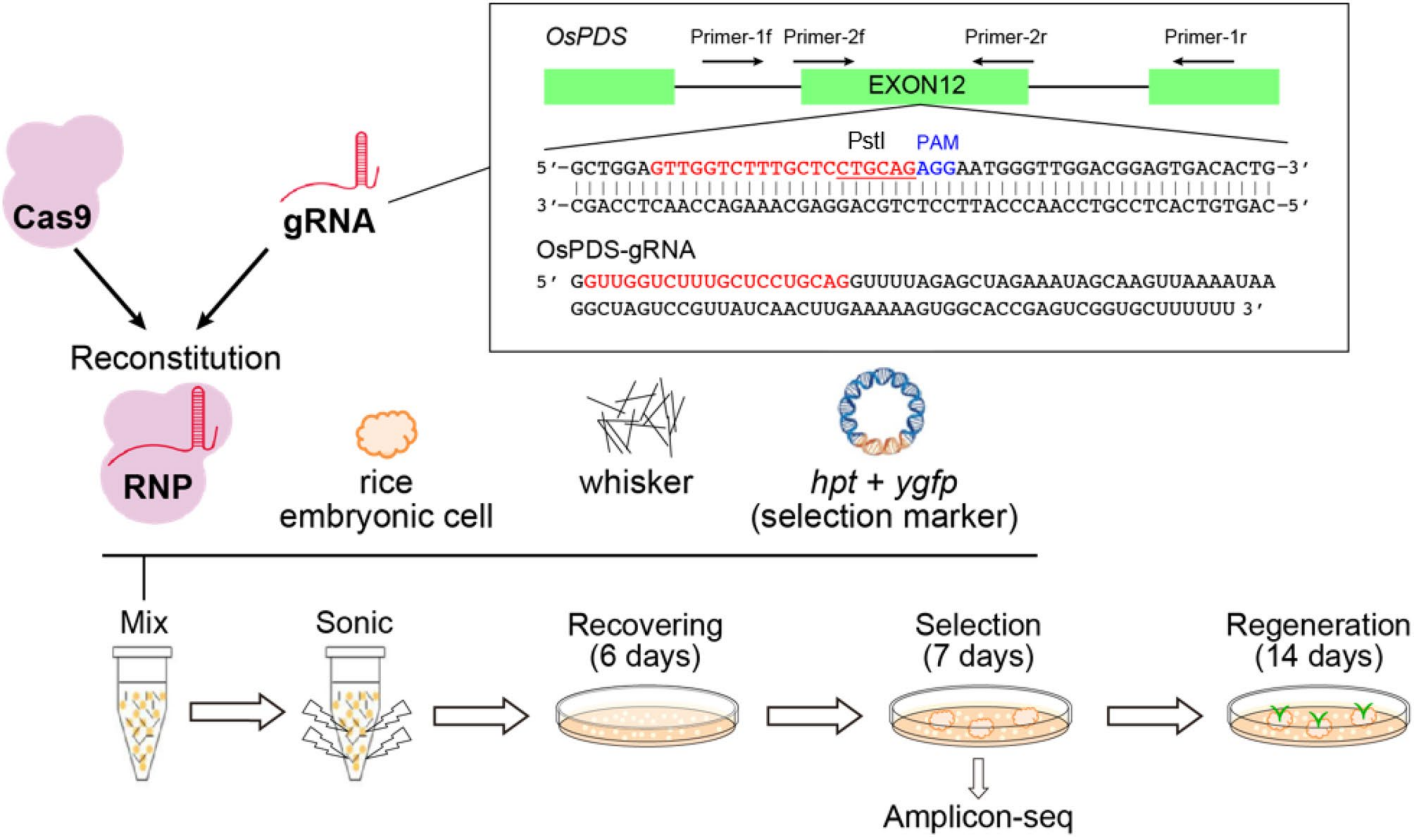
Overview of the experimental procedure of CRISPR/Cas9 RNP delivery with the sonication-assisted whisker method using selection marker-harboring plasmids. The recombinant SpCas9-NLS and in vitro transcribed gRNA were mixed to prepare the RNPs. The RNPs were mixed with the whiskers, the selection marker-harboring, plasmids, and specific amounts of rice embryonic suspension cells (250 packed cell volume [PCV]). The calli mixed with whiskers and RNPs were ultrasonicated by a sonicator. Sonication-treated cells were washed with the R2 medium and incubated without antibiotics for the recovery culture. After the 6-day recovery culture, we conducted a hygromycin-based selection of the transformed calli for 1–2 weeks. Selected calli were split into fragments, one of which was subjected to DNA extraction and genome sequence analysis using Amplicon sequencing (Amplicon-seq). The rest of the selected calli, not used for genome DNA analyses, were incubated further and transferred into a regeneration medium to obtain the shoot. *OsPDS* exon12 was selected as the genome editing target (inlet). The target sequence of the gRNA and the PAM sequence were marked as red and blue, respectively. The restriction enzyme PstI recognition site is underlined. The in vitro transcribed gRNA sequence is also indicated in the inlet.

First, we attempted RNP delivery into rice embryonic cell suspensions using a selection marker (Fig. 1). The *OsPDS* locus was selected as a genome editing target site using a gRNA reportedly efficient for genome editing^21^. For the large-scale RNP preparation, we purified mg-scale recombinant SpCas9 fused with a nuclear localization signal (NLS) and performed in vitro gRNA synthesis (Supplementary Fig. 1a,b). The activity of the RNPs, consisting of SpCas9-NLS and the gRNA targeting *OsPDS,* was assessed using an in vitro cleavage assay (Supplementary Fig. 1c). We confirmed that nearly 90% of the substrate DNA could be digested by the prepared RNPs during a 30-min incubation at 37 °C. Next, we mixed the RNPs with the potassium titanate whiskers and the plasmid harboring both hygromycin phosphotransferase (*HPT)* and *Chiridius poppei Yellowish-Green Fluorescent Protein (CpYGFP)* expression cassettes^22^, and subjected them to sonication with rice cell suspensions according to the protocol we established previously^17^. For RNP delivery optimization, we tested several RNP concentrations (Table 1). We washed the whisker-treated calli with the R2 medium and incubated them without any antibiotics for the recovery culture. After the 6-day recovery culture, we conducted a hygromycin-based selection of the transformed calli for 1–2 weeks. The selected calli were split into fragments, one of which was subjected to DNA extraction and genome sequence analysis using Amplicon sequencing (Amplicon-seq). The rest of the selected calli, not used for genome DNA analyses, were incubated further and transferred into a regeneration medium to obtain a shoot.

**Table 1:** Summary of *OsPDS* genome editing experiments in rice by the sonication-assisted whisker method.

RNP pmol/250 PCV	Number of hyg selected T0 calli	Number of selected calli with the expected phenotype	Number of calli with mutation/total number of calli genotyped	Number of the mutants with biallelic mutations/number of calli with mutations
0	16	0	0	–
2	8	1	0/8	–
20	28	9	0/28	–
40	3	1	1/3	1/1
100	23	16	9/22	3/9
Plasmid	21	10	10/21	8/10

Summary of three independent experiments. Detailed genotypes are shown in Supplementary Table 1.

*hyg* hygromycin.

**Figure 2:**
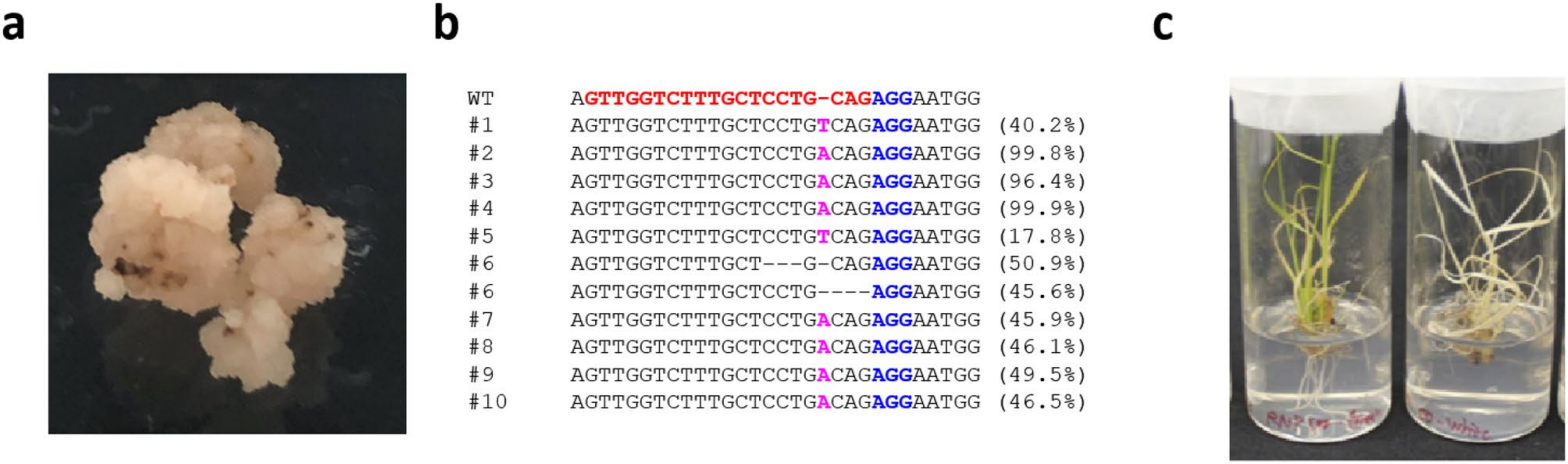
Representative results of *OsPDS* locus genome editing in rice by RNP delivery using the sonication-assisted whisker method. (**a**) A representative image of white-colored calli after the incubation of the selection medium for 2 weeks; (**b**) genome sequences of the *OsPDS* with targeted mutations in the whisker-treated calli after the incubation of the selection medium for 2 weeks. DNA from independently selected calli were analyzed by Amplicon-seq. The gRNA target sequence and the PAM sequence are colored in red and blue, respectively. The insertion bases are highlighted in magenta. The numbering on the left corresponds to independent calli (e.g., #6 has two types of mutations in one callus). The genotypes are also summarized in Supplementary Table 1. The percentages in the right of the sequences indicate the percentage of the reads with mutation/the reads with no mutations; (**c**) a representative image of regenerated plants with green- (control, left), and white-colored shoots (genome-edited, right).

During the selection, we obtained several white-colored calli with phenotypes identical to that of the *OsPDS* mutant, and detected the mutations at the target sites in the genome of the calli (Fig. 2a). Several calli could be selected by the hygromycin harbor 1-bp insertion at the putative DSB site in the *OsPDS*, indicating successful genome editing events by the RNPs (Fig. 2b). Related to the RNP concentration, the highest investigated concentration, 100 pmol/250 packed cell volume (PCV), was proven the most efficient for genome editing (9 out of 22 selected calli, Table 1). The genome editing efficiency was similar to that of the conventional plasmid delivery approaches using the sonication-assisted whisker methods (10 out of 20 selected calli, Table 1). These results suggest that 100 pmol RNPs/250 PCV is a sufficient concentration to produce genome-edited lines in rice embryonic cell suspensions using the sonication-assisted whisker method. The low ratio of isolating mosaic mutant in 100 pmol RNPs/250 PCV suggests that genome editing events occurred just after the whisker treatments in the small number of whisker-inserted cells (Supplementary Table 1). Notably, the Amplicon-seq results showed that the 1-bp insertion is dominant in the patterns of the RNP-induced mutations, whereas > 10-bp deletions frequently occurred in the mutants produced by plasmids even though the gRNA sequence used in the case of the two conditions was identical (Supplementary Table 1, Supplementary Fig. 2). Although the difference was not statistically significant, this tendency implies that transient RNP delivery induces smaller-size mutations compared with the plasmid delivery, in which case continuous RNP production and resultant continuous mutation induction would occur. Consistently, the biallelic mutant ratio in the genome-edited lines was lower in the RNP-delivered lines than that in the plasmid-delivered lines (Table 1, Supplementary Table 1). The regeneration from the RNP-delivered calli occurred normally, resulting in albino individuals in certain cases (Fig. 2c). Although the *OsPDS* mutant exhibited growth termination, we could anticipate the induced mutation inheritance in the case of non-lethal genes.

### Targeted mutagenesis of lycopene cyclase genes in rice

**Figure 3:**
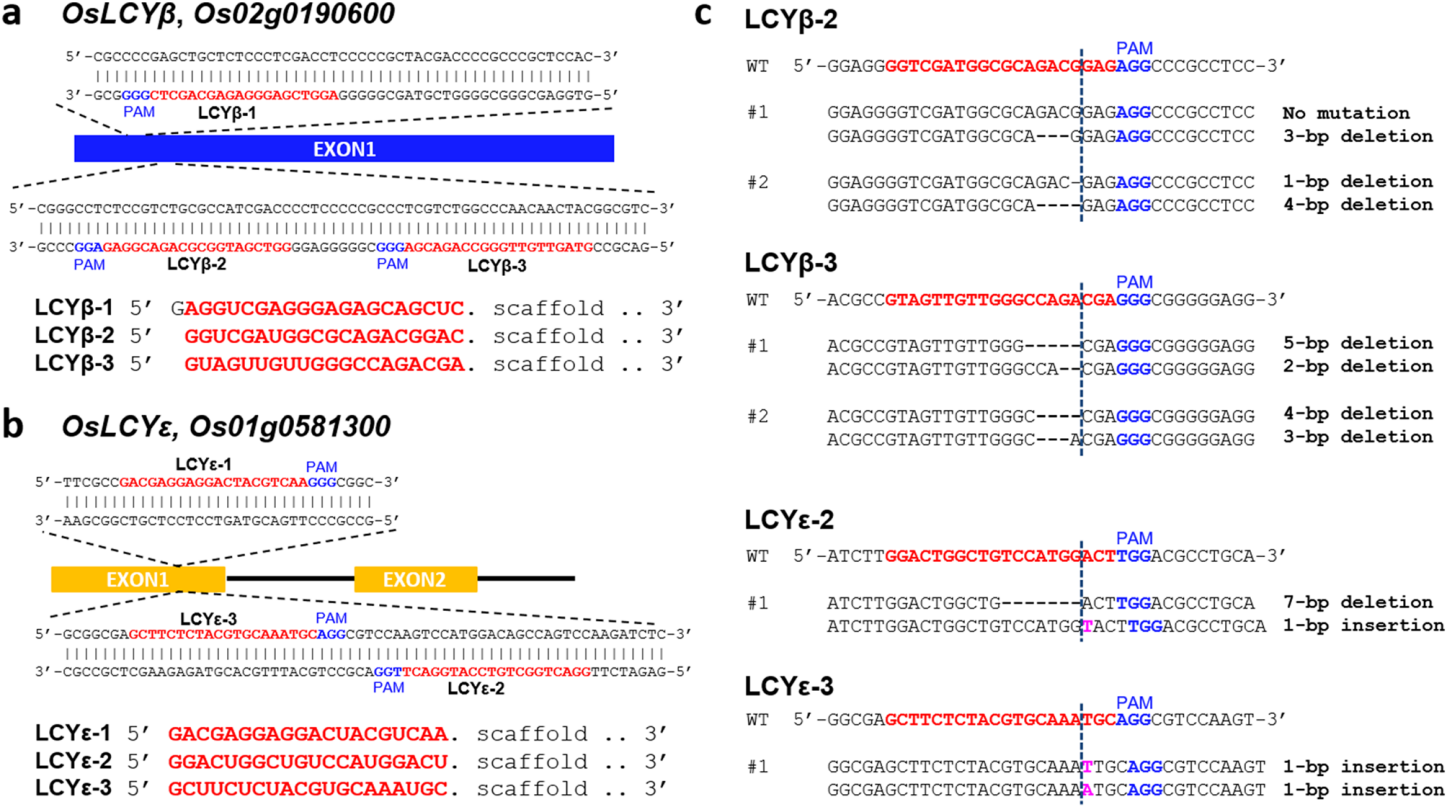
Isolation of *OsLCYβ* and *OsLCYε* mutants using RNP delivery by the sonication-assisted whisker method. (**a**) gRNA design for *OsLCYβ* genome editing*.* *OsLCYβ* exon1 was selected as the genome editing target. The target sequences of each gRNA and the PAM sequence are marked in red and blue, respectively. The in vitro transcribed gRNA sequence is also indicated; (**b**) gRNA design for *OsLCYε* genome editing*. OsLCYε* exon1 was selected as the genome editing target. The coloration of the schematic image is the same as those of (**a**). **(c)** Detailed mutations detected in the selected calli of the *OsLCYβ* or *OsLCY* *ε* mutants. The name of gRNAs is indicated in each sequence alignment. The genome sequence with no mutation is described as “WT.” The insertions and the PAM sequence are highlighted in magenta and blue, respectively. The gRNA target sequence is colored with red. Dashed lines indicate the putative DSB sites.

Next, we attempted to assess the sonication-assisted whisker method with the optimized conditions for RNPs applicable for functional genetics, by challenging to isolate genome-edited lines of the genes with mutants not reported in rice. We selected *LYCOPENE CYCLASE BETA* (*OsLCYβ, Os02g0190600*) and *LYCOPENE CYCLASE EPSILON* (*OsLCYε, Os01g0581300*) as the target genes for genome editing (Fig. 3a,b). A previous study implies that the expression level reduction of these genes results in rice with high lycopene content^23^, although no single gene knockouts of these genes have been reported so far. We designed three gRNAs for *OsLCYβ* or *OsLCYε*. The activity of each gRNA was assessed by in vitro cleavage assays (Supplementary Fig. 3). The in vitro cleavage assay revealed that most RNPs with these gRNAs, LCY*β*-1, LCY*β*-2, LCY*β*-3, and LCY*ε*-3, exhibited higher activities compared to that with the gRNA targeting *OsPDS* (Supplementary Fig. 3), suggesting that highly efficient genome editing could be expected when using RNPs with the aforementioned gRNAs. We transfected RNPs together with the plasmid harboring *HPT* and *CpYGFP* to the rice calli under the 100 pmol/250 PCV condition (Table 2). The hygromycin-selected calli were subjected to Amplicon-seq analyses and certain selected lines exhibited mutations at the target sites (Fig. 3c, Tables 1, 2). However, the genome editing efficiency was not higher than that in the case of *OsPDS* disruption. Mutants with biallelic mutations in each gene were also isolated (two out of four selected calli in LCY*β*-2, two out of three in LCY*β*-3, one LCY*ε*-2, and one LCY*ε*-3). These results indicate that the sonication-assisted-whisker-based RNP delivery could be used for the isolation of the genome-edited lines of genes other than *OsPDS*. Unexpectedly, no genome editing events occurred under certain conditions using RNPs with the gRNA with higher or similar activity in vitro as the gRNA for *OsPDS* (Supplementary Fig. 3, Table 2, e.g., LCY*β*-1, and LCY*ε*-1). The discrepancy between the gRNA activity in vitro and in vivo is frequently reported^24^, and it is usually thought to derive from the cell selection, propagation, and DNA repair status complexity in the target cells. In addition, there is a possibility that gRNA in vitro cleavage efficiency is correlated with genome editing efficiency in calli at higher concentration condition > 100 pmol/250 PCV. This could also be the case for the sonication-assisted whisker method since obtaining the selected calli took 2–3 weeks.

**Table 2:** Summary of the genome editing experiments of *OsLCYβ* and *OsLCYε* in rice by RNP delivery with the sonication-assisted whisker method.

gRNA	Number of hyg selected T0 calli	Number of T0 calli with mutations/total calli genotyped	Rate of the lines with biallelic mutations/T0 calli with mutations	Number of callus with pigment
LCY*β*-1	19	0	0	0
LCY*β*-2	24	4/24	2/4	2
LCY*β*-3	7	3/7	2/3	1
LCY*ε*-1	7	0	0	0
LCY*ε*-2	5	1/5	1/1	0
LCY*ε*-3	4	1/4	1/1	0

Summary of three independent experiments.

*hyg* hygromycin.

### Analyses of the genome-edited *OsLCYβ* line

**Figure 4:**
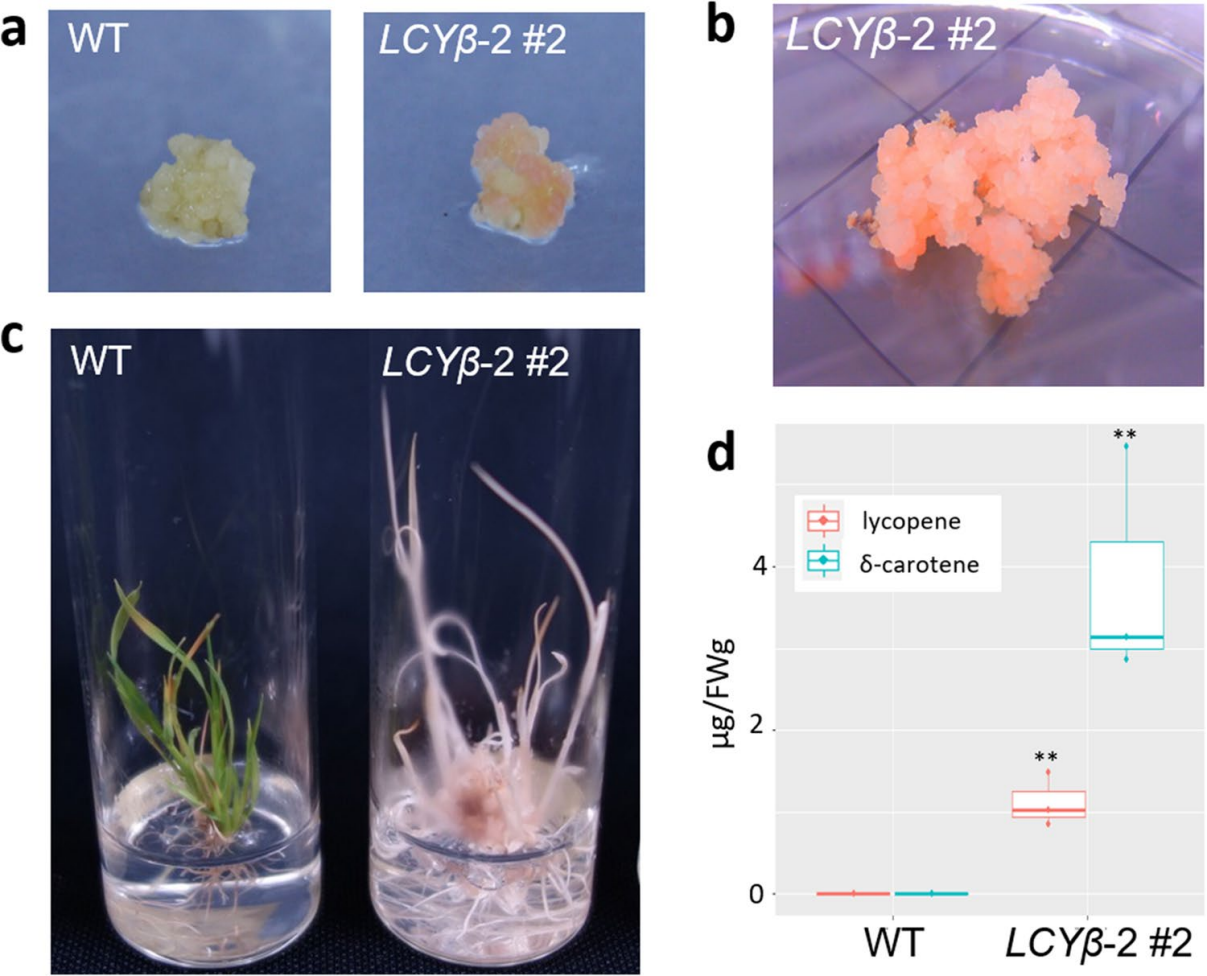
Phenotypic analysis of the *OsLCYβ* mutant generated by RNP delivery using the sonication-assisted whisker method. (**a**) Representative images of non-RNP-treated calli (left) and genome-edited lines of *LCYβ*-2 #2 after the 2-week selection (right); (**b**) the callus of *LCYβ*-2 #2 showed clear red pigmentation; (**c**) representative images of the shoot from the non-RNP-treated calli (left) and that from the genome-edited calli of *LCYβ*-2 #2; (**d**) quantification of the carotenoids in the *LCYβ*-2 #2 calli, **indicate statistical significance analyzed by the Welch Two Sample t-test between WT and *LCYβ*-2 #2 (*p* < 0.05).

In the isolated genome-edited *OsLCYβ* lines, we observed that certain calli had red pigments (Fig. 4a,b). In the *LCYβ*-2 #2 mutant, we obtained a regenerated plant with abnormal pigmentation (Fig. 4c). Since LCY*β* catalyzes δ-carotene transition to α-carotene as well as trans-lycopene to γ-carotene, its knock-out mutations possibly induce δ-carotene accumulation, resulting in yellow–red color and the presence of trans-lycopene^25^. To assess carotenoid accumulation in the mutant, we conducted liquid chromatography (LC) with a photodiode array (PDA) and electrospray mass spectrometer (MS), LC-PDA-MS analyses (Fig. 4d). In the callus of the *LCYβ*-2 #2 mutant, as expected, δ-carotene was markedly accumulated compared with the non-RNP-treated control calli, accumulating almost no δ-carotene (Fig. 4, Supplementary Fig. 4). In addition to δ-carotene, we also detected lycopene accumulation in the callus. Consistently, lycopene with red color is the upstream substrate of δ-carotene in the carotenoid biosynthesis pathway^23^. *OsLCYβ* disruption could induce not only δ-carotene accumulation but also that of upstream substrates. Callus pigmentation could be explained by both lycopene and δ-carotene accumulation. However, carotenoid accumulation in the regenerated shoot from the callus of the *LCYβ*-2 #2 mutant was not similar to that in the callus (Supplementary Fig. 5). Clear reductions of β-carotene, lutein, violaxanthin, and antheraxanthin in the *LCYβ*-2 #2 mutant plantlet could be detected. It was consistent that these carotenoids were downstream of *OsLCYβ* in the carotenoid biosynthesis pathway*.* Notably and unexpectedly, we identified lutein acetate accumulation during our MS/MS analyses in the *LCYβ*-2 #2 mutant plantlet. The *LCYβ*-2 mutants did not develop their flowers and displayed growth termination, suggesting that *OsLCYβ* is an essential gene in rice. In summary, these results demonstrated that RNP delivery by the sonication-assisted whisker method can be used for functional genetics in rice.

### Selection marker-free RNP delivery-based genome editing in rice

All the above-described genome editing experiments were conducted using RNPs with the plasmid harboring selection markers. Due to CRISPR-Cas9 RNP complex has affinity to double strand DNA^1^ and can bind with plasmid DNA, it is not obvious that RNPs can be delivered by whisker method without addition of any carrier DNAs. Accordingly, we tried *OsPDS*-target RNP delivery by the sonication-assisted whisker method without plasmid co-transfection and any selections (Supplementary Fig. 6). We did not obtain any callus with a white-color phenotype in the pilot experiment of this approach, indicating a significantly lower genome-edited than non-edited cell population. To detect the rare genome editing events, a cleaved amplified polymeric sequences assay was used (see “Methods” section in detail). We performed Amplicon-seq analyses of the PstI-digested PCR products, amplified from the genome DNA of the calli harvested 2 weeks after the RNP transfection. We observed six out of 36 samples harboring the 1-bp insertion or deletion mutations at the target site while the samples treated with no RNPs never displayed such mutations (Supplementary Fig. 6). This result suggests that the sonication-assisted whisker method could deliver RNPs to induce genome editing at the target site in rice embryonic cell suspensions without plasmid co-transfection.

## Discussion

In this study, we determined that the condition of successful RNP delivery using the sonication-assisted whisker method in rice embryonic suspension cells is 100 pmol RNPs/250 PCV (Table 1). We isolated several biallelic mutants using 100 pmol RNPs/250 PCV conditions with hygromycin selection (Figs. 2, 3). In the conventional plasmid delivery using the sonication-assisted whisker method, 20 µg plasmids/125–250 PCVs were used for isolating 10–20 transgenic lines^17^. Therefore, 100 pmol RNPs per one condition should be highly sufficient in common usage for isolating genome-edited lines in rice. It is noteworthy that the RNP amount for the 100 pmol/condition using the sonication-assisted whisker method is comparable to that of the PEG or the particle bombardment methods^9, 26, 27^. This suggests that the sonication-assisted whisker method is a practical approach to delivering CRISPR RNPs to rice calli.

The insertion or deletion (indels) sizes induced by the RNP delivery mainly remained under 10 bp, whereas those induced by the plasmid delivery were beyond 10 bp in this study (Figs. 2, 3, Supplementary Table 1, Supplementary Fig. 2). Continuous Cas9 and gRNA expression by the plasmids could potentially induce DSBs multiple times even after the target site carried small indels since the CRISPR/Cas9 system could cleave the target DNA sequence with insertion or deletion and change its mutations repeatedly^28^. RNP delivery possibly induces smaller indels at the target site. This characteristic is preferable to induce small indels, which mimic naturally-occurring mutations.

Unlike T-DNA delivery by *Agrobacterium*, gene delivery methods achieved by physical principles, including particle bombardment and the whisker method, could induce the fragmentation of the DNAs to be delivered^6^. The larger the DNA fragment to be delivered is, the possibility of fragmented DNA random integration becomes higher. Therefore, the size of the plasmid to be delivered should be as small as possible for transgene elimination. In the case of CRISPR/Cas9-based genome editing, the large-size DNA fragments for expressing Cas9 and gRNA are usually problematic^29^. Delivery of both the small-size plasmid harboring the selection marker and CRISPR/Cas9 components as RNPs reduces the risk of random integration of the transgene into the genome. Unfortunately, the isolation of null segregant has not been achieved in this study since the target genes were lethal. Establishing procedures and assessment of the efficiency for isolating null segregants should be further challenges for the whisker mediated RNP delivery method. The feasible isolation of genome-edited lines by the sonication-assisted whisker method in this study would contribute to developing a platform of transgene-free genome-edited lines.

*OsLCYβ* genome-edited rice was first developed by the sonicated-assisted whisker method with RNPs (Figs. 3, 4, Supplementary Figs. 4, 5). Drastic reduction of β-carotene, violaxanthin, antheraxanthin were observed in the plantlet of the *OsLCYβ* gene-edit line. Chlorophylls may not be maintained without carotenoids since the adequate carotenoid:chlorophyll ratio is essential for the function and maintenance of the photosynthetic reaction center complex^30^, thereby leading to the albino phenotype^31^. In addition to lycopene and δ-carotene accumulation in the callus, lutein acetate was unexpectedly accumulated and other carotenoids were reduced in the regenerated shoot of the *OsLCYβ* mutant. Lutein acetate accumulation was also reported in rice leaves during senescence, possibly due to the degradation of light-harvesting complex II^32^. Different from lutein, lutein acetate cannot be part of the photosynthetic reaction center complex^32^. Adequate carotenoid shortage in the photosynthetic reaction center complex could explain why the shoot of the *LCYβ*-2 #2 mutant, which cannot synthesize the carotenoids for photosynthesis, displays no green color.

This study is the first to report that RNPs could be delivered into plant cells by the whisker method. In particular, RNPs were probably introduced without plasmid co-transfection (Supplementary Fig. 6). The results suggest that the sonication-assisted whisker method could be used not only to conduct RNP delivery but also to deliver macromolecules other than plasmids. Although the detailed underlying mechanism of how RNPs entered into plant cells has not been clarified in this study, the sonication-assisted whisker method could be potentially used to introduce proteins into plant cells in general. Unlike particle bombardment, RNPs were probably introduced into the plant cells in solution without any dry-up process, thereby protein delivery with less inactivation could be achieved. For example, as a future perspective, recombinant antibiotic resistance proteins co-transfected with CRISPR/Cas RNPs could make DNA-free genome-edited cell selection possible.

The sonication-assisted whisker method has already been used as a versatile method for gene delivery in plants^14–18^. The demonstration of RNP-based genome editing in rice in this study could easily be extrapolated to various plant species. The sonication-assisted whisker method would be an attractive tool for RNP-based gene delivery not only in the model crops but also in industrially important plant species.

## Methods

### Recombinant SpCas9 preparation

The C-terminally His_6_-tagged SpCas9 with SV40 NLS gene was cloned into pET28a (Addgene #47327^33^). For recombinant SpCas9-NLS overexpression, the plasmid was transformed into the *E. coli* strain BL21 (DE3) pLysRARE2. The bacteria containing the plasmid were pre-cultured with the supplementation of 25 μg/mL kanamycin and 34 μg/mL chloramphenicol. When the OD_600_ reached 0.6, 0.25 mM IPTG was added to induce protein expression at 18 °C overnight. Cell pellets were resuspended in buffer A [50 mM Tris–HCl, pH 8.0, 500 mM NaCl, 5 mM MgCl_2_, and 10% (vol/vol) glycerol] with 0.5 mg/mL lysozyme and 0.1 mg/mL DNase I, then disrupted by sonication. The supernatant was loaded onto a 1-mL Ni–NTA Superflow column pre-equilibrated with buffer A. The recombinant SpCas9 protein was eluted with 250 mM imidazole, then two-times diluted with buffer B [50 mM Tris–HCl, pH 8.0, 5 mM MgCl_2_, and 10% (vol/vol) glycerol]. The diluted sample was loaded onto a Heparin HP column. The protein fractions were pooled and finally purified by a HiLoad 16/600 Superdex 200 pg column in buffer C [20 mM Tris–HCl pH 7.5, 150 mM NaCl, 1 mM MgCl_2_, and 10% (vol/vol) glycerol]. Pooled fractions were concentrated by ultrafiltration to a final concentration of 100 μM and stored at − 80 °C until further use.

### In vitro gRNA synthesis

The gRNA was transcribed using a lab-made T7 RNA polymerase. The double-strand DNA encoding the T7 promoter and gRNA sequence was amplified by PCR using four overlapping primers [F1: 5′-TAATACGACTCACTATANNNNNNNNNNNNNNNNNNNNNGTTTTAGAGCTAGAAATAGCAAGTTAAAATAAG-3′ (N means 20-bp target sequence dependent on the target gene, see Supplementary Table 1 for further details); F2: 5′-GGATCCTAATACGACTCACTATAG-3′; R1: 5′-GCACCGACTCGGTGCCACTTTTTCAAGTTGATAACGGACTAGCCTTATTTTAACTTGCTATTTCTAGCTC-3′; R2: 5′-AAAAAAGCACCGACTCGGTGCCAC-3′] and purified using a MinElute PCR Purification Kit (QIAGEN Inc.). The in vitro transcription was performed at 37 °C for 6 h. The reaction mixture was purified using 8% denaturing urea-polyacrylamide gel electrophoresis. RNAs were extracted from the gel slices and refolded simultaneously in H_2_O at 4 °C for 18 h. The extracted RNA samples were precipitated with ethanol, dissolved in H_2_O, and stored at − 80 °C.

### In vitro gRNA activity assessment

Target site-containing substrate DNA fragments were PCR-amplified using rice genomic DNA as a template. The amplified DNA fragment (10 nM) was mixed with the recombinant SpCas9–sgRNA complex (10–500 nM, molar ratio, 1:2) in 10 μL of Cas9 reaction buffer (20 mM HEPES–NaOH, pH 7.5, 100 mM NaCl, 5 mM MgCl_2_, and 0.1 mM EDTA), then incubated at 37 °C for 30 min. The reaction mixture was quenched by adding 10 μL of EDTA (50 mM final concentration)-containing quench buffer, then incubated at 60 °C for 5 min. The reaction products were analyzed using a MultiNA microchip electrophoresis system (SHIMADZU Inc.). For the time course experiment of the in vitro cleavage assay, the amplified DNA fragment (10 nM) was incubated at 25 °C or 37 °C with the recombinant SpCas9–sgRNA complex (100 nM, molar ratio, 1:2), in 30 μL of Cas9 reaction buffer. Aliquots (10 μL) were taken at 5 and 30 min and mixed with 10 μL of quench buffer, then incubated at 60 °C for 5 min. The reaction products were analyzed using a MultiNA microchip electrophoresis system. In vitro cleavage assays were performed at least three times.

### Plasmid preparation

The plasmid containing the selective marker *HPT* (hygromycin phosphotransferase) gene and *CpYGFP* (a yellow GFP-like protein derived from the marine copepod *Chiridius poppei*) controlled by the 35S promoter was purified by a QIAGEN plasmid Maxi Kit (QIAGEN Inc.). The *OsPDS*-targeted CRISPR/Cas9 vector for the sonication-assisted whisker method was constructed using pZH_gYSA_PubiMMCas9 and pU6_ccdB_gRNA^34^.

### Cell culture

Calli were induced from mature rice seed (*Oryza sativa* L. ssp. Japonica cv. Nipponbare) and suspension-cultured as reported previously^35^. After suspension culture for 3 weeks, the cell clumps were crushed and passed through a one-mm stainless sieve immediately prior to use. The cell preparation is identical both in “with selection” experiment or “without selection” experiments.

### Whisker preparation

Whiskers of potassium titanate fibers (Whisker LS20; TITAN KOGYO Inc.) with an average diameter of 0.5 nm and length ranging between 5 and 30 nm were used for transformation. Approximately 50 mg of dry fibers into tube were sterilized with 1 mL EtOH (100%) overnight and dried by leaving the tube with an opened cap on the clean bench. The sterilized whiskers were stored at 4 °C until use. The dry whiskers were handled in an exhaust fume hood to avoid inhalation. Sterile whiskers in liquid medium (1% w/v) containing 1/3MS basal salts and 30 g/L sucrose were mixed using a vortex mixer.

### Transformation, selection, and regeneration

Approximately 250 µL in PCV of suspension-cultured rice calli after a 3-day subculture were dispensed into a 1.5-mL Eppendorf tube. The calli were dispersed in a fresh liquid medium containing 1/3 MS salts and 30 g/L sucrose. The 1% (w/v) whisker suspension (500 µL), vortexed immediately before use, was added to this tube and tapped for mixing. After centrifugation at 3000 rpm for 5 s, the supernatant was removed. The plasmid/polyornithine (POH) complex (70 µL) harboring antibiotics-resistant gene cassette was mixed with ice-cold 0–100 pmol RNPs in the tube (i.e., in case of 100 pmol RNP, 1 µM in final) and the tube was tapped strongly to mix except for the DNA-free experiments. For the DNA-free experiments, only the RNP with POH of 100 µL was mixed with the whisker. Immediately, the suspension was centrifuged for 5 min at 15,000 rpm at 4 °C and tapped. This process was repeated 3 times. After incubation on ice for 10 min, the tube containing the mixture of calli, whiskers, and the plasmid was subjected to supersonic treatment with a disrupter for 1 min (Bioruptor UCD-200, Sonicbio. Co. Ltd.) at room temperature. Next, the mixture was washed with a fresh liquid medium to remove the whiskers. The whisker-treated calli were then transferred into a 35 × 10 mm plastic Petri plate for subculture. Three ml of liquid R2 medium (1 × R2 basal salts, 2 mg/L 2, 4-D, and 30 g/L sucrose). The culture was grown on a rotary shaker (120 rpm) in the dark at 28 °C.

After 3–6 days, the whisker-treated calli were transferred onto the surface of the selection medium [N6 basal salts, 2 mg/L 2, 4-D, 30 g/L sucrose, 50 mg/L hygromycin B (Hyg), and 3 g/L Gelrite (Wako Chemicals Inc.)]. After 7 days of culture, the calli were manually split into pieces and subjected to DNA extraction (see below). After 30 days of culture, the growing calli were transferred into a regeneration medium (MS basal salts, 1 mg/L NAA, 2 mg/L BA, 20 g/L sucrose, 30 g/L sorbitol, 50 mg/L Hyg, and 4 g/L Gelrite) and cultured at 28 °C with 16 h of light per day.

### PCR genotyping and Amplicon-seq analysis

DNA was extracted from the part of calli using the cetyltrimethylammonium bromide (CTAB) method. The target region was PCR-amplified using KOD One PCR master Mix or KOD FX Neo (TOYOBO Inc., Osaka, Japan) with the primer set of the first-round PCR (see Supplementary Table 1). PCR was carried out using the cycling conditions as follows: 98 °C for 2 min followed by 35 cycles of 98 °C for 10 s, 55 °C for 5 s, and 68 °C for 3 s. In the experiment of DNA-free genome editing, the restriction enzyme which recognizes the putative DSB site by CRISPR/Cas9 were utilized for first round PCR product. Since the DNA fragments with certain mutations at the target site should be resistant to the restriction enzyme, the genome-edited DNA fragments would be concentrated in the PCR product upon the treatment with the restriction enzyme. The PCR products were digested by PstI-HF (New England Biolab Inc., Massachusetts, USA) and gel-purified using FastGene Gel/PCR Extraction Kit (Nippon Genetics Inc., Tokyo, Japan) to reduce unedited Os*PDS* amplicons (See also Supplementary Fig. 6). In the second-round PCR, Illumina Combinatorial Dual Index sequences were attached as follows: 5′-AATGATACGGCGACCACCGAGATCTACACNNNNNNNNTCGTCGGCAGCGTC-3′ and 5′-CAAGCAGAAGACGGCATACGAGATNNNNNNNNGTCTCGTGGGCTCGG-3′. PCR was carried out using the cycling conditions as follows: 98 °C for 2 min followed by 30 cycles of 98 °C for 10 s, 55 °C for 5 s, and 68 °C for 3 s. The PCR products were purified using 1.2 times-volume of Sera-Mag Select beads (Cytiva Inc., Marlborough, MA, USA) with a supernatant substituted with 20% PEG-8000 and 2.5 M NaCl solution. The equal volume of purified DNA was pooled and its concentration was measured using a Qubit dsDNA HS Assay Kit and Qubit 3.0 Fluorometer (Thermo Fisher Scientific Inc., Waltham, MA, USA) and adjusted to 50 pM. The pooled library was used for 2 × 150-bp paired-end sequencing with iSeq100 (Illumina Inc., San Diego, CA, USA) after mixing with 0.25 volume of 50 pM PhiX control v3 (Illumina Inc.). The obtained fastq files were analyzed by CRISPResso2^36^. Indels at the Cas9 cleavage site were considered as mutations induced by the introduced RNPs. The mutation quantification window was set as 10 bp.

### Carotenoid identification and quantification

We performed carotenoid extraction from the calli (0.5 g) and shoots (0.25 g) with biological triplicates following a previously reported procedure^23^. The acetone supernatants of the extracts were dried and the samples were kept at − 20 °C. Right before analysis, the dried sample was resuspended in 1 mL acetone with 0.1% butylated hydroxytoluene (BHT). An aliquot (2.0–2.5 µL) of this sample was injected into a UPLC system (described below) and the carotenoids were identified by UV–Vis, MS, and MS/MS spectral data and by retention time in HPLC and compared to standard samples. Authentic β-carotene, lutein, and neoxanthin were purchased from Fujifilm Wako (#032-17991, #524-31401, and #523-31471, respectively). Standards for ε-carotene, δ-carotene, lycopene, and violaxanthin were prepared by extraction from recombinant *E. coli* producing each carotenoid.

The instrument used was a Waters ACQUITY UPLC H-Class PLUS System with Xevo TQ-S micro equipped with an electrospray ionization source operating in positive mode. Carotenoid separation was performed on a BEH UPLC C18 column (100 mm × 2.1 mm, 1.7 μm) (Waters Inc., Milford, CT) at 30 °C. The mobile phases consisted of solvents A (water:acetonitrile 10:90), B (100% acetonitrile), and C (acetonitrile:isopropanol 60:40) using the following gradient elution program at a flow rate of 0.2 mL/min: 0–2 min, 100% A; 2–4 min, gradient to 100% B; 4–6 min, gradient to 100% C; 6.01 min, back to 100% A, and hold for 3 min. The injection volume was 2–2.5 µL. The desolvation gas was set to 1000 L/h at 300 °C, the cone gas flow was at 50 L/h. The capillary voltage was set to 3.5 kV and the cone voltage at 30 V for all samples. The MS/MS experiments were performed using a collision energy of 20 V. The data were analyzed using the MassLynx 4.1 software equipped to the instrument. The carotenoid amounts were quantified as previously described^37, 38^ by calculating the UPLC peak area at 450 nm using the standard curve of authentic samples.

### Data availability

Quantified carotenoid data were analyzed and visualized using R version 4.1.1 (R Core Team). The R script used for the data analyses and source data are available on Github (https://github.com/aist-pgrrg/whisker_RNP).

### Supplementary Information

Supplementary Information.
