# Supplementary material for: The sonication-assisted whisker method enables CRISPR-Cas9 ribonucleoprotein delivery to induce genome editing in rice
Source: Sci Rep. 2023 Sep 7;13:14205. doi: 10.1038/s41598-023-40433-w (PMC10484913; doi:10.1038/s41598-023-40433-w)

**Supplementary Figures**

**
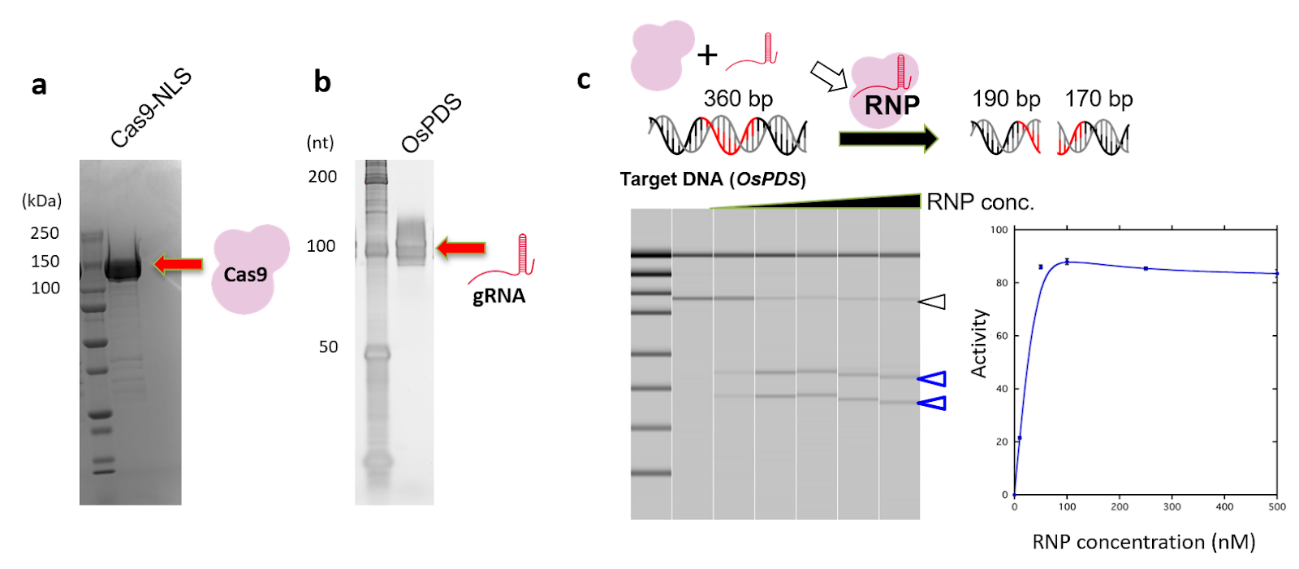
**

**Supplementary Fig. 1:** ***In vitro* SpCas9-gRNA RNP preparation.**

**a** SDS-PAGE image showing the recombinant SpCas9-NLS protein purity; **b** The Urea-PAGE image of the *in vitro* transcribed gRNA targeting the *OsPDS* gene; **c** *In vitro* cleavage assay using the recombinant RNPs as an enzyme and the PCR product of *OsPDS* as a substrate. The upper drawing shows the schematic image of the *in vitro* cleavage assay. The pseudo-electrophoresis image was reconstituted from the electrophoresis data from the output of capillary electrophoresis, which analyzed the products of the *in vitro* cleavage reaction (left). The DNA digestion activity, calculated by signals from undigested products / all the signals (see Methods in detail), are highlighted on the right.

| Condition | Genotype |  | Frequency |
| --- | --- | --- | --- |
| 40 pmol/PCV | 3 bp del/3 bp del | Transhetero | 1 |
| 100 pmol/PCV | T ins | Homo | 1 |
| 100 pmol/PCV | T ins | Mosaic | 1 |
| 100 pmol/PCV | A ins | Homo | 3 |
| 100 pmol/PCV | A ins | Hetero | 4 |
| plasmid | 54 bp del | Homo | 1 |
| plasmid | A ins/38 bp del | Transhetero | 2 |
| plasmid | 8 bp del | Homo | 1 |
| plasmid | 4 bp del | Homo | 1 |
| plasmid | G ins | Homo | 1 |
| plasmid | A ins | Homo | 1 |
| plasmid | T ins | Homo | 1 |
| plasmid | 1 bp del | Hetero | 1 |

**Supplementary Table S1: List of all the mutations in genome-edited calli in OsPDS produced by the sonication-assisted whisker method.** The "Condition" column indicates the RNP concentration or plasmid used. The "Genotype" column describes the mutation rate in the amplicon: "Homo" indicates a mutation rate >95% with a single mutation type, "Hetero" indicates a mutation rate between 45% and 55% with a single mutation type, "Transhetero" indicates a sample with two types of mutations and a total mutation rate >95%, and "Mosaic" indicates a mutation rate <30% with a single mutation type. The "Frequency" column refers to the number of independently isolated genome-edited calli (Related to Table 1, Figure 2b).


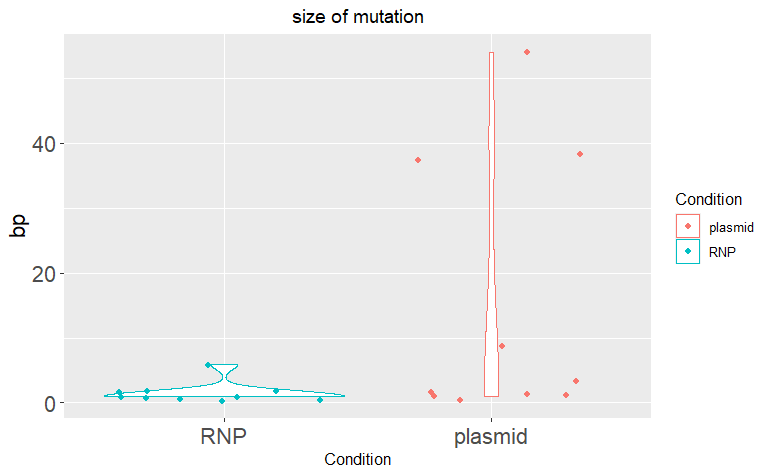


**Supplementary Fig. 2: Difference of insertion/deletion size in genome-edit callis between RNP delivery and plasmid delivery.** Plots of length insertion or deletion size in two conditions. Plots showing the length of insertions or deletions in the two conditions. The distribution difference between the two conditions was tested using the Kruskal-Wallis rank sum test (*P* = 0.0611). Detailed data can be found in Supplementary Table 1.

***
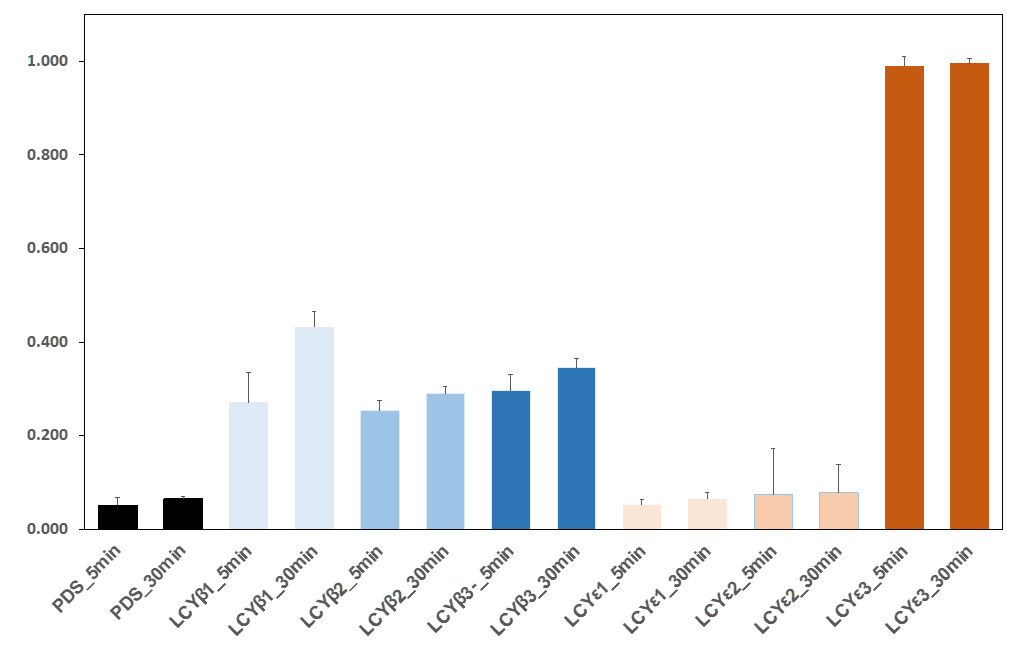
***

**Supplementary Fig. 3: *in vitro* cleavage assay of the gRNAs used in this study.**

The time course experiments of the *in vitro* cleavage assay were performed at 25°C. Each gRNA was mixed with the recombinant SpCas9, then incubated with the substrate DNA fragment for 5 and 30 min. The *in vitro* cleavage assays were performed at least three times.


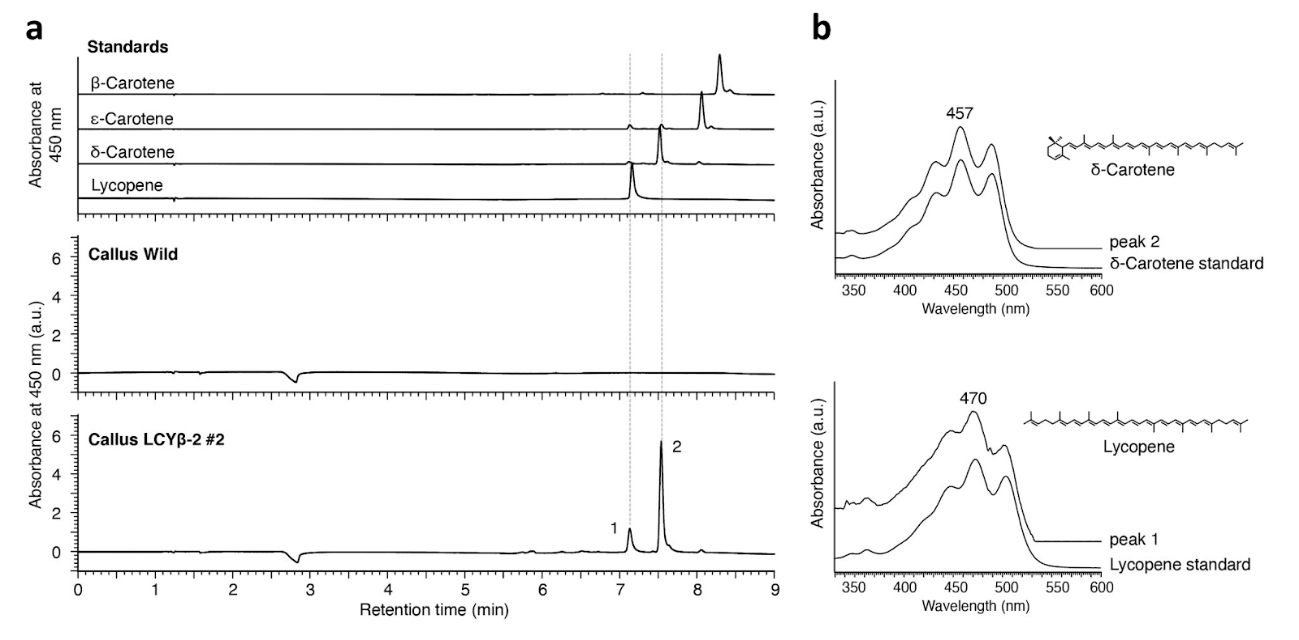


**Supplementary Fig. 4:** **LC-PDA-MS analysis of the callus extract of the *LCYβ-2* #2 mutant. a** PDA chromatogram of the carotenoid standards (top) and the extracted samples (bottom). The peak area from this chromatogram was used to quantify the carotenoids, shown in main Figure 4d; **b** The absorbance spectrum of peak 1 or 2 identified in the chromatogram of callus *LCYβ-2* #2 in panel **a**. Peaks 1 and 2 were identified as lycopene and δ-carotene, respectively, based on the retention time, absorbance spectrum, and mass spectrum.


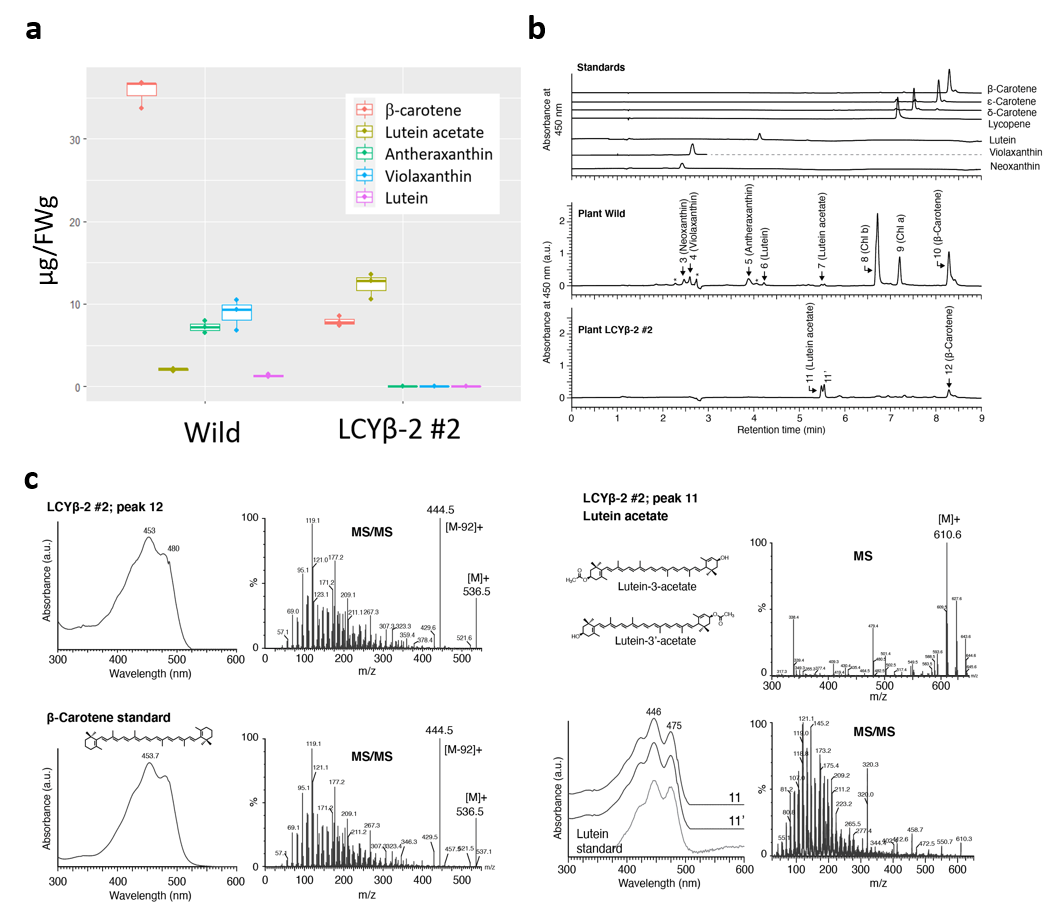


**Supplementary Fig. 5: LC-PDA-MS/MS analyses of the extract from the shoot of the *LCYβ-2* #2 mutant.** **a** Carotenoid production of the plant of the Wild or the *LCYβ-2* #2 mutant. The amount was calculated using the peak area of the PDA chromatogram indicated in the panel. Each box plot indicates the quantification data of three samples from three different calli derived from the *LCYβ-2* #2 lines. Quantities of all the types of measured carotenoids exhibited a significant difference between the Wild and the *LCYβ-2* #2 mutant (Wilcoxon rank-sum test, *p* < 0.01); **b** Representative chromatogram of the carotenoid extracts; **c** Absorbance (left) and MS/MS (right) spectra of peaks 11, 11’, and 12 from the chromatogram of plant *LCYβ-2* #2 extract in panel **b**. Peak 12 is identified as β-carotene since its absorbance spectrum, MS/MS spectrum, and retention time matched with the authentic standard of β-carotene. For peaks 11 and 11’, the molecular mass was 610, which is 42 mass heavier than that of lutein, and the absorbance spectrum was identical to that of lutein. Consistent with a previous study (Kusaba et al. 2009), the retention time was between that of lutein (4.2 min) and chlorophyll b (6.7 min). Based on these results and the fact that lutein 3’-acetate was detected in rice leaves (Kusaba et al. 2009), the carotenoids of peak 11 and 11’ are most probably either lutein 3-acetate or lutein 3’-acetate.


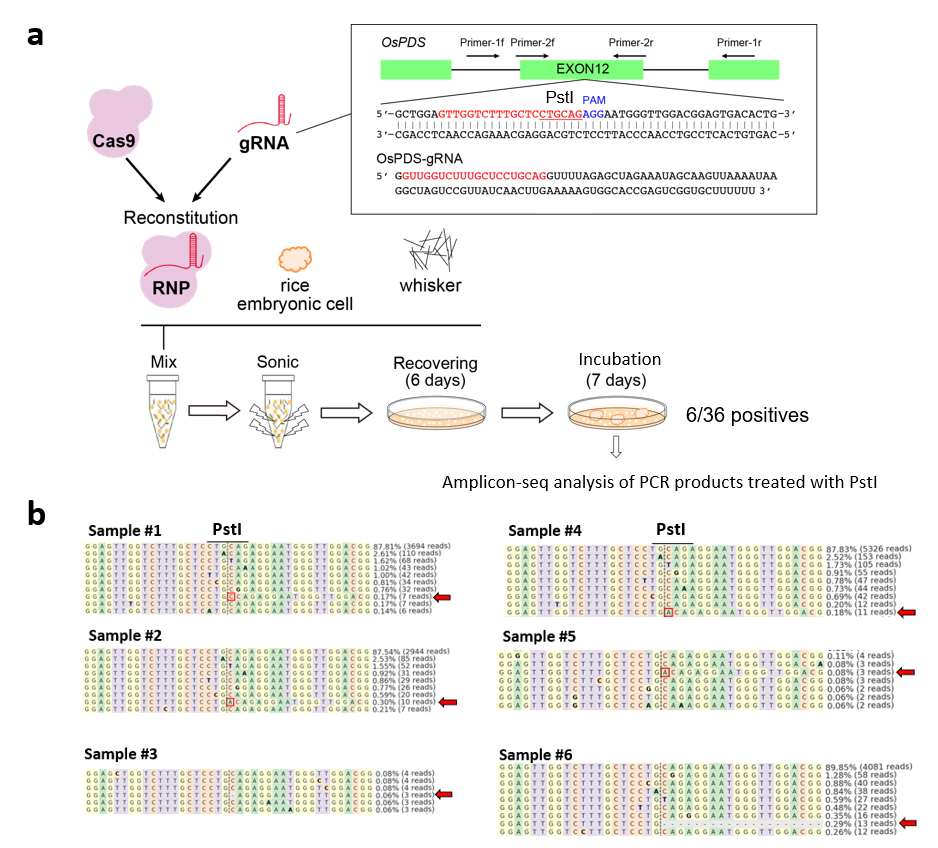


**Supplementary Fig. 6: Genome editing using the whisker-mediated RNP delivery without additional DNA**

**a** Overview of the experimental procedure (see also Fig.1). The recombinant SpCas9-NLS and *in vitro* transcribed gRNA were mixed to form RNPs. The RNPs were mixed with the whiskers, selection marker-harboring plasmids, and specific amounts of rice embryonic suspension cells (250 PCV). The calli mixed with the whiskers and RNPs were ultrasonicated by a sonicator. Sonication-treated cells were washed with the R2 medium and incubated without antibiotics for the recovery culture. After the 6-day recovery culture, we conducted a subsequent 1-week incubation. The propagated calli were subjected to DNA extraction and the genome sequence was analyzed by Amplicon-seq. Six out of 36 samples carried mutations. *OsPDS* exon12 was selected as the genome editing target (inlet). The target sequence of the gRNA for *OsPDS* and the PAM sequence are marked in red and blue, respectively. The restriction enzyme PstI recognition site is underlined. The *in vitro* transcribed gRNA sequence is also indicated in the inlet; **b** The mutations detected in the six samples are shown sample by sample. The results of Amplicon-seq were summarized by CRISPResso2. The wild-type sequence is the uppermost. The reads with mutations are indicated by red arrows.


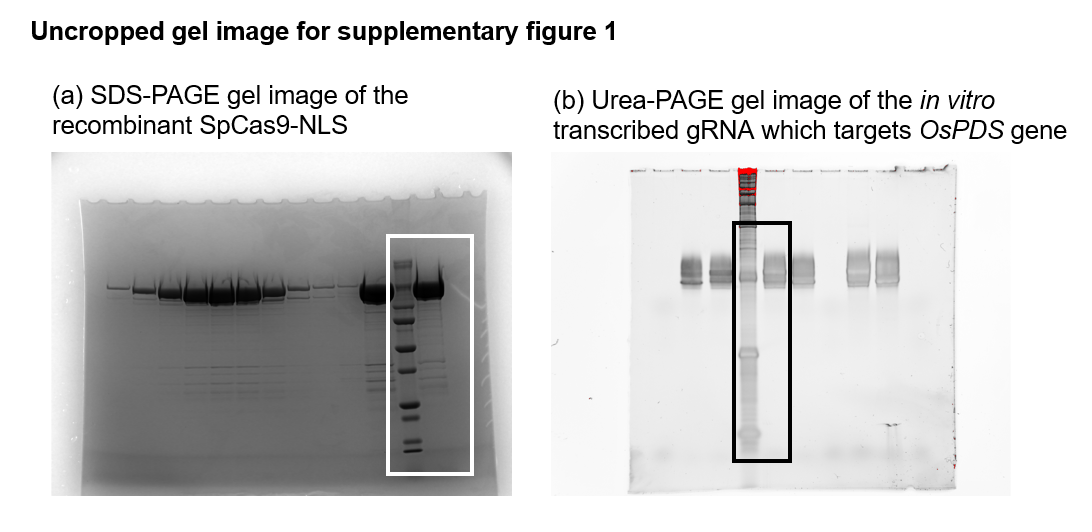

Supplement: Supplementary file 1 — Supplementary Information. [file 41598_2023_40433_MOESM1_ESM.docx]
